# Supplementary material for: Analysis of Fatty Acid Composition and Volatile Profile of Powder from Edible Crickets (Acheta domesticus) Reared on Apple By-Products
Source: Foods. 2024 May 26;13(11):1668. doi: 10.3390/foods13111668 (PMC11172300; doi:10.3390/foods13111668)
Supplement: Supplementary file 1 [file foods-13-01668-s001.zip › foods-2970250-supplementary_20240508.pdf]

Table S1. List of reagents used.

Used reagent	Company	Purity
Chloroform	Kanto Chemical Co., Inc. (Tokyo, Japan)	99.7 %
Methanol	Kanto Chemical Co., Inc. (Tokyo, Japan)	99.8 %
Potassium chloride	FUJIFILM Wako Pure Chemical Corporation (Osaka, Japan)	99.5 %
Anhydrous sodium sulfate	FUJIFILM Wako Pure Chemical Corporation (Osaka, Japan)	-
Potassium hydroxide	FUJIFILM Wako Pure Chemical Corporation (Osaka, Japan)	85.0 %
Sulfuric acid	FUJIFILM Wako Pure Chemical Corporation (Osaka, Japan)	95.0 %
n-Hexane	Kanto Chemical Co., Inc. (Tokyo, Japan)	96.0 %
Methyl nonadecanoate	Tokyo Chemical Industry Co., Ltd. (Tokyo, Japan)	98.0 %
Supelco 37 Component FAME Mix	Supelco Inc. (Bellefonte, PA, USA)	-
Ethanol	Kanto Chemical Co., Inc. (Tokyo, Japan)	99.5 %
D(+)-Glucose	Kanto Chemical Co., Inc. (Tokyo, Japan)	98.0 %
D(-)-Fructose	FUJIFILM Wako Pure Chemical Corporation (Osaka, Japan)	99.0 %
Sucrose	FUJIFILM Wako Pure Chemical Corporation (Osaka, Japan)	99.0 %
Maltose monohydrate	Kanto Chemical Co., Inc. (Tokyo, Japan)	98.0 %
Trehalose	HAYASHIBARA CO., LTD. (Okayama, Japan)	98.0 %
Sodium carbonate	FUJIFILM Wako Pure Chemical Corporation (Osaka, Japan)	99.8 %
Folin & Ciocalteu's Phenol Reagent	FUJIFILM Wako Pure Chemical Corporation (Osaka, Japan)	-
Gallic Acid Monohydrate	Tokyo Chemical Industry Co., Ltd. (Tokyo, Japan)	98.0 %

All reagents were analytical grade.

Table S2. Fatty acid contents of diets used in this study (mg/kg DM).

Shorthand Nomenclature	Fatty Acid Name	Type	CD	ABPs
12:0	Lauric acid	SFA	136.5 ± 35.3	55.0 ± 12.6 *
14:0	Myristic acid	SFA	305.2 ± 123.0	14.3 ± 3.4
14:1 (*n*-5)	Myristoleic acid	MUFA	14.8 ± 4.5	n.d.
15:0	Pentadecanoic acid	SFA	26.1 ± 6.4	n.d.
16:0	Palmitic acid	SFA	5750.8 ± 1280.2	1330.2 ± 148.5 *
16:1 (*n*-7)	Palmitoleic acid	MUFA	386.9 ± 97.9	8.9 ± 2.7 *
17:0	Margaric acid	SFA	65.2 ± 14.1	15.6 ± 3.3 *
18:0	Stearic acid	SFA	1914.7 ± 368.0	359.5 ± 17.4 *
18:1 (*n*-9)	Oleic acid	MUFA	9557.5 ± 2120.2	3261.5 ± 372.9 *
18:1 (*n*-9)	Elaidic acid	MUFA	27.1 ± 7.8	n.d.
18:2 (*n*-6)	Linoleic acid	PUFA	8314.7 ± 1646.8	5286.5 ± 188.4
18:3 (*n*-6)	γ-Linolenic acid	PUFA	711.0 ± 148.6	166.1 ± 8.6 *
18:3 (*n*-3)	α-Linolenic acid	PUFA	161.1 ± 89.5	44.9 ± 3.2
20:1 (*n*-9)	Eicosenoic acid	MUFA	54.0 ± 18.4	121.0 ± 16.0 *
20:2 (*n*-6)	Eicosadienoic acid	PUFA	4.2 ± 0.8	7.2 ± 1.3 *
20:3 (*n*-6)	Eicosatrienoic acid	PUFA	47.0 ± 6.8	n.d.
20:3 (*n*-3)	Eicosatrienoic acid	PUFA	25.3 ± 4.4	34.5 ± 3.3 *
20:4 (*n*-6)	Arachidonic acid	PUFA	389.1 ± 74.9	n.d.
20:5 (*n*-3)	Eicosapentaenoic acid	PUFA	33.0 ± 4.2	32.9 ± 1.1
21:0	Heneicosanoic acid	SFA	19.7 ± 2.7	12.3 ± 1.4 *
22:0	Behenic acid	SFA	8.0 ± 1.0	n.d.
22:1 (*n*-9)	Erucic acid	MUFA	6.3 ± 2.1	n.d.
22:6 (*n*-3)	Docosahexaenoic acid	PUFA	767.6 ± 135.3	n.d.
C23	Tricosanoic acid	SFA	11.4 ± 2.5	n.d.
C24	Lignoceric acid	SFA	n.d.	14.8 ± 4.1
24:1 (*n*-9)	Nervonic acid	MUFA	66.1 ± 11.7	n.d.
	Total		28803.1 ± 6145.6	10765.3 ± 710.2 *
	Σ SFAs		8237.5 ± 1819.0	1801.7 ± 129.1 *
	Σ MUFAs		10112.8 ± 2244.9	3391.4 ± 385.0 *
	Σ PUFAs		10452.9 ± 2086.7	5572.1 ± 202.5
	Σ *n*-3 PUFAs		986.9 ± 222.4	112.3 ± 7.0 *
	Σ *n*-6 PUFAs		9466.0 ± 1864.4	5459.8 ± 195.6
	Σ *n*-6/*n*-3		9.6 ± 0.3	48.6 ± 1.3 *

Values are expressed as means ± standard deviations for powder from crickets reared in separate cages (n = 3). Powder from crickets from the same cage was measured in triplicate. * indicates significant differences between samples ($p < 0.05$). CD: control diet; ABPs: apple by-products; SFAs: saturated fatty acids; MUFAs: monounsaturated fatty acids; PUFAs: polyunsaturated fatty acids; n.d.: not detected; DM: dry matter.

Table S3. Volatile profiles of diets used in this study.

Volatile Compounds	CD	ABPs
Aldehydes		
2-Butenal, 2-ethyl-	0.10 ± 0.03	n.d.
2-Butenal, 2-methyl-	0.09 ± 0.01	n.d.
Benzeneacetaldehyde	0.03 ± 0.00	n.d.
Butanal, 2-methyl-	0.41 ± 0.02	3.54 ± 0.25 *
Butanal, 3-methyl-	0.73 ± 0.03	0.85 ± 0.09
Heptanal	0.58 ± 0.03	0.11 ± 0.03 *
Hexanal	9.77 ± 0.88	2.08 ± 0.16 *
Nonanal	0.63 ± 0.03	n.d.
Pentanal	1.37 ± 0.13	0.71 ± 0.10 *
Propanal	0.14 ± 0.03	n.d.
Propanal, 2-methyl-	0.11 ± 0.01	0.44 ± 0.03 *
2-Butenal, 3-methyl-	n.d.	0.02 ± 0.00
2-Heptenal, (E)-	n.d.	0.52 ± 0.07
2-Pentenal, (E)-	n.d.	0.02 ± 0.00
Acetaldehyde	n.d.	0.77 ± 0.06
Total	13.95 ± 1.10	9.21 ± 0.71 *
Ketones		
1-Pentanone, 1-(4-methylphenyl)-	0.01 ± 0.00	n.d.
2-Heptanone	0.22 ± 0.01	0.01 ± 0.00 *
3,5-Octadien-2-one	0.23 ± 0.02	n.d.
Acetoin	0.23 ± 0.05	0.22 ± 0.02
Ethanone, 1-(2,3-dihydro-1H-inden-5-yl)-	0.01 ± 0.00	n.d.
1,2-Cyclopentanedione	n.d.	0.11 ± 0.02
2,3-Pentanedione	n.d.	0.36 ± 0.03
2-Propanone, 1-hydroxy-	n.d.	1.06 ± 0.12
4-Cyclopentene-1,3-dione	n.d.	0.25 ± 0.04
Nonane, 3-methyl-5-propyl-	n.d.	2.82 ± 0.20
Nonane, 5-butyl-	n.d.	0.04 ± 0.05
Total	0.71 ± 0.03	4.88 ± 0.02 *
Hydrocarbon		
2-Octene, 2,6-dimethyl-	0.25 ± 0.00	n.d.
5-Ethyldecane	0.13 ± 0.00	n.d.
5-Undecene, 5-methyl-	0.16 ± 0.01	n.d.
Cyclopentane, methyl-	0.29 ± 0.10	n.d.
Cyclopropane, 1-(2-methylbutyl)-1-(1-methylpropyl)-	0.43 ± 0.01	n.d.
Decane	10.63 ± 0.26	n.d.
Decane, 5-propyl-	0.03 ± 0.00	n.d.
Dodecane	4.80 ± 0.56	n.d.
Dodecane, 4,6-dimethyl-	0.10 ± 0.02	n.d.
Dodecane, 4-methyl-	0.05 ± 0.01	n.d.
Heptane, 2,4-dimethyl-	0.27 ± 0.05	n.d.
Heptane, 4-ethyl-	0.13 ± 0.02	n.d.
Hexadecane	0.05 ± 0.02	n.d.
Nonane, 2,2,4,4,6,8,8-heptamethyl-	0.88 ± 0.12	n.d.

Nonane, 2,5-dimethyl-	0.32 ± 0.02	n.d.
Nonane, 2-methyl-	0.29 ± 0.01	n.d.
Nonane, 5-propyl-	0.25 ± 0.07	n.d.
Octane, 2,6,6-trimethyl-	0.70 ± 0.01	2.30 ± 2.40
Octane, 3,3-dimethyl-	0.44 ± 0.03	n.d.
Octane, 4-methyl-	0.25 ± 0.01	n.d.
Pentadecane	0.09 ± 0.01	n.d.
Pentadecane, 2,6,10,14-tetramethyl-	0.05 ± 0.00	n.d.
Pentane, 3-methyl-	0.08 ± 0.04	n.d.
Tetradecane	0.18 ± 0.15	n.d.
Tridecane, 6-methyl-	0.11 ± 0.01	n.d.
Undecane, 2,5-dimethyl-	0.11 ± 0.01	n.d.
Undecane, 3,8-dimethyl-	0.26 ± 0.01	0.27 ± 0.05
Undecane, 5-methyl-	0.30 ± 0.03	n.d.
Undecane, 6,6-dimethyl-	0.03 ± 0.01	0.76 ± 0.33
Undecane, 6-ethyl-	0.03 ± 0.01	n.d.
1,1,3,3,5-Pentamethylcyclohexane	n.d.	0.16 ± 0.02
1-Decene, 2,4-dimethyl-	n.d.	0.06 ± 0.01
1-Pentadecene	n.d.	0.04 ± 0.00
2,2,4,4-Tetramethyloctane	n.d.	6.01 ± 0.31
2,5-Cyclohexadiene, 1,4-diethyl-1,4-dimethyl-	n.d.	0.00 ± 0.00
3-Ethyl-3-methylheptane	n.d.	0.81 ± 0.39
5-Hepten-2-one, 6-methyl-	n.d.	0.67 ± 0.09
7-Tetradecene	n.d.	0.02 ± 0.00
Cyclododecane	n.d.	0.11 ± 0.02
Cyclohexane, (1,3-dimethylbutyl)-	n.d.	0.05 ± 0.00
Decane, 2-cyclohexyl-	n.d.	0.00 ± 0.00
Decane, 3,3,8-trimethyl-	n.d.	0.39 ± 0.16
Decane, 3,6-dimethyl-	n.d.	0.28 ± 0.02
Decane, 3-ethyl-3-methyl-	n.d.	0.04 ± 0.01
Decane, 5-ethyl-5-methyl-	n.d.	0.09 ± 0.01
Dodecane, 2,6,10-trimethyl-	n.d.	2.02 ± 0.69
Heptane, 3,3,4-trimethyl-	n.d.	0.23 ± 0.01
Octane, 2,2,6-trimethyl-	n.d.	0.03 ± 0.01
Octane, 2,6-dimethyl-	n.d.	1.61 ± 0.40
Octane, 2-cyclohexyl-	n.d.	0.02 ± 0.00
Octane, 6-ethyl-2-methyl-	n.d.	0.77 ± 0.33
Pentane, 2,2,4,4-tetramethyl-	n.d.	0.08 ± 0.01
Tridecane	n.d.	0.02 ± 0.00
Undecane, 3,6-dimethyl-	n.d.	0.88 ± 0.18
Undecane, 5,7-dimethyl-	n.d.	0.26 ± 0.08
Total	21.70 ± 0.86	17.99 ± 3.87
Benzenoids		
Benzene, 1,2,4-trimethyl-	0.17 ± 0.00	0.03 ± 0.00 *
Benzene, 1,3-dimethyl-	0.26 ± 0.01	0.07 ± 0.00 *
Benzene, 1-ethynyl-4-methyl-	0.05 ± 0.00	n.d.
Benzene, 1-methyl-3-(1-methylethyl)-	1.70 ± 0.06	n.d.
Benzene, 1-methyl-4-propyl-	0.03 ± 0.00	n.d.

Benzenepropanenitrile	0.12 ± 0.01	n.d.
Ethylbenzene	0.23 ± 0.04	0.06 ± 0.01 *
1,1'-Biphenyl, 2,2',5,5'-tetramethyl-	n.d.	0.00 ± 0.00
Benzene, 1,3-bis(1-methylethenyl)-	n.d.	0.00 ± 0.00
Benzene, 1,4-diethyl-	n.d.	0.02 ± 0.00
Butylated Hydroxytoluene	n.d.	1.40 ± 0.24
Phenol, 4-(1,1-dimethylpropyl)-	n.d.	0.12 ± 0.02
Total	2.56 ± 0.06	1.70 ± 0.25 *
Pyrazines		
Pyrazine, 2,5-dimethyl-	0.19 ± 0.01	n.d.
Pyrazine, 3-ethyl-2,5-dimethyl-	0.05 ± 0.00	n.d.
Pyrazine, methyl-	0.11 ± 0.01	n.d.
Total	0.35 ± 0.02	n.d.
Pyridine		
Pyridine	0.06 ± 0.00	n.d.
Pyridine, 2-ethyl-	0.04 ± 0.00	n.d.
Total	0.10 ± 0.00	n.d.
Pyran		
4H-Pyran-4-one, 2,3-dihydro-3,5-dihydroxy-6-methyl-	n.d.	1.63 ± 0.41
Total	n.d.	1.63 ± 0.41
Furan		
Furan, 2-ethyl-	0.15 ± 0.03	n.d.
Furan, 2-pentyl-	0.55 ± 0.01	n.d.
2,2'-Bifuran	n.d.	0.02 ± 0.00
2,4-Dihydroxy-2,5-dimethyl-3(2H)-furan-3-one	n.d.	0.45 ± 0.06
2-Acetyl-2-methyltetrahydrofuran	n.d.	0.05 ± 0.01
2-Furancarboxaldehyde, 5-methyl-	n.d.	0.91 ± 0.06
3-Furaldehyde	n.d.	42.87 ± 2.59
3-Furanmethanol	n.d.	1.93 ± 0.35
5-Methyl-2-(2-methyl-2-tetrahydrofuryl)tetrahydrofuran	n.d.	0.22 ± 0.02
Ethanone, 1-(2-furanyl)-	n.d.	1.19 ± 0.07
Furan, 3-methyl-	n.d.	0.06 ± 0.01
Total	0.70 ± 0.03	47.71 ± 2.67 *
Sulfur compounds		
Bis-(methylthio)-phosphine	0.15 ± 0.00	n.d.
Disulfide, dimethyl	0.38 ± 0.09	n.d.
Sulphuric acid dibutyl ester	0.14 ± 0.02	n.d.
Dimethyl sulfide	n.d.	0.19 ± 0.03
Total	0.67 ± 0.07	0.19 ± 0.03 *
Acids		
Acetic acid	17.51 ± 0.56	7.33 ± 0.57 *
Butanoic acid, 2-methyl-	0.54 ± 0.02	0.03 ± 0.00 *
Butanoic acid, 3-methyl-	1.29 ± 0.27	n.d.
Butanoic acid, 4-hydroxy-	0.20 ± 0.01	0.16 ± 0.02
Hexanoic acid	0.18 ± 0.01	n.d.
Propanoic acid	1.62 ± 0.41	n.d.

Propanoic acid, 2,2-dimethyl-	0.31 ± 0.02	n.d.
Propanoic acid, 2-methyl-	0.53 ± 0.04	n.d.
Acetic acid, (acetyloxy)-	n.d.	0.32 ± 0.04
Benzoic acid	n.d.	0.22 ± 0.05
Formic acid	n.d.	0.17 ± 0.04
Propanoic acid, 2-methyl-, anhydride	n.d.	1.68 ± 0.16
Total	22.16 ± 0.70	9.90 ± 0.74 *
Amines		
(2-Aziridinylethyl)amine	0.48 ± 0.03	n.d.
Pyrrolidine	n.d.	0.07 ± 0.01
Total	0.48 ± 0.03	0.07 ± 0.01 *
Terpenes		
(+)-4-Carene	0.14 ± 0.02	n.d.
.alpha.-Phellandrene	0.06 ± 0.00	n.d.
.gamma.-Terpinene	0.81 ± 0.03	n.d.
3-Carene	0.43 ± 0.01	0.03 ± 0.00 *
Bicyclo[3.1.0]hex-2-ene, 4-methyl-1-(1-methylethyl)-	0.12 ± 0.00	n.d.
D-Limonene	28.41 ± 1.54	n.d.
Thymol	0.05 ± 0.00	n.d.
.alpha.-Farnesene	n.d.	0.35 ± 0.02
Longifolene	n.d.	0.02 ± 0.01
Total	30.01 ± 1.53	0.40 ± 0.02 *
Esters		
Acetic acid, phenyl-, isopentyl ester	0.03 ± 0.01	n.d.
d-Proline, N-methoxycarbonyl-, heptyl ester	0.09 ± 0.00	n.d.
Octanoic acid, ethyl ester	0.17 ± 0.03	n.d.
Pentanoic acid, 5-hydroxy-, 2,4-di-t-butylphenyl esters	0.02 ± 0.01	0.02 ± 0.00
Propanoic acid, 2-hydroxy-, ethyl ester, (S)-	0.66 ± 0.06	n.d.
Propanoic acid, 2-methyl-, 3-hydroxy-2,4,4-trimethylpentyl ester	0.01 ± 0.00	n.d.
1,2-Ethanediol, diacetate	n.d.	0.22 ± 0.02
1-Butanol, 2-methyl-, acetate	n.d.	0.37 ± 0.02
2,2-Dimethylpropanoic anhydride	n.d.	1.32 ± 0.11
Acetic acid ethenyl ester	n.d.	0.42 ± 0.03
Acetic acid, butyl ester	n.d.	0.12 ± 0.01
Acetic acid, methyl ester	n.d.	0.07 ± 0.01
Butanoic acid, 2-methyl-, hexyl ester	n.d.	0.21 ± 0.01
Butanoic acid, hexyl ester	n.d.	0.06 ± 0.02
Butanoic acid, propyl ester	n.d.	0.01 ± 0.00
Hexanoic acid, hexyl ester	n.d.	0.03 ± 0.02
Total	0.98 ± 0.08	2.85 ± 0.04 *
Lactones		
2(3H)-Furanone, 5-butyldihydro-	0.08 ± 0.00	n.d.
2(3H)-Furanone, dihydro-5-pentyl-	0.06 ± 0.01	n.d.
Hydrazinecarboxamide	n.d.	0.45 ± 0.02
2(3H)-Furanone, 5-methyl-	n.d.	0.09 ± 0.03
2(5H)-Furanone	n.d.	0.20 ± 0.02

Total	0.14 ± 0.01	0.73 ± 0.07 *
Ethers		
Methylal	0.11 ± 0.01	n.d.
Oxetane, 3-(1-methylethyl)-	0.19 ± 0.02	n.d.
Total	0.29 ± 0.01	n.d.
Alcohols		
1-Hexanol	0.39 ± 0.05	n.d.
1-Octen-3-ol	0.47 ± 0.04	n.d.
1-Pentanol	1.09 ± 0.04	n.d.
1-Penten-3-ol	2.30 ± 0.05	n.d.
1-Butanol, 2-methyl-	n.d.	0.53 ± 0.04
1-Decanol, 2-ethyl-	n.d.	1.58 ± 0.38
1-Pentanol, 4-methyl-	n.d.	0.26 ± 0.01
2-Propanol, 1-methoxy-	n.d.	0.15 ± 0.01
Total	4.24 ± 0.18	2.52 ± 0.33 *
Others		
Hydrazinecarboxamide	0.96 ± 0.07	n.d.
2-Cyclohexen-3-ol-1-one, 2-[1-iminoethyl]-	n.d.	0.03 ± 0.00
Methane, isocyanato-	n.d.	0.15 ± 0.02
Tetradecyl trifluoroacetate	n.d.	0.04 ± 0.01
Total	0.96 ± 0.07	0.23 ± 0.02 *

Values are expressed as means ± standard deviations for powder from crickets reared in separate cages (n = 3). Powder from crickets from the same cage was measured in quintuplicate. * indicates significant difference between powders (p < 0.05). CD: control diet; ABPs: apple by-products; n.d.: not-detected.
